# Wavefront-Guided versus Non-Wavefront-Guided Photorefractive Keratectomy for Myopia: Meta-Analysis of Randomized Controlled Trials

**DOI:** 10.1371/journal.pone.0103605

**Published:** 2014-07-29

**Authors:** Hidenaga Kobashi, Kazutaka Kamiya, Keika Hoshi, Akihito Igarashi, Kimiya Shimizu

**Affiliations:** 1 Department of Ophthalmology, University of Kitasato School of Medicine, Kanagawa, Japan; 2 Department of Preventive Medicine, University of Kitasato School of Medicine, Kanagawa, Japan; Bascom Palmer Eye Institute, University of Miami School of Medicine, United States of America

## Abstract

**Purpose:**

To compare the efficacy, predictability, safety, and induced higher-order aberrations (HOAs) between wavefront-guided and non-wavefront-guided photorefractive keratectomy (PRK).

**Methods:**

The Cochrane Central Register of Controlled Trials, PubMED, and EMBASE were searched for randomized controlled trials. Trials meeting the selection criteria were quality appraised, and data was extracted by 2 independent authors. Measures of association were pooled quantitatively using meta-analytical methods. Comparisons between wavefront-guided and non-wavefront-guided ablations were made as pooled odds ratios (ORs) or weighted mean differences. The pooled ORs and 95% confidence intervals (CIs) were computed for efficacy, safety, and predictability. The weighted mean differences and 95% CIs were used to compare induced HOAs.

**Results:**

The study covered five trials involving 298 eyes. After wavefront-guided PRK, the pooled OR of achieving an uncorrected distance visual acuity of 20/20 (efficacy) was 1.18 (95% CI, 0.53–2.60; p = 0.69), the pooled OR of achieving a result within ±0.50 diopter of the intended target (predictability) was 0.86 (95% CI, 0.40–1.84; p = 0.70). No study reported a loss of 2 or more lines of Snellen acuity (safety) with either modality. In eyes with wavefront-guided PRK, the postoperative trefoil aberrations (mean difference −0.02; 95% CI, −0.03 to −0.00; p = 0.03) were significantly lower. There were no significant differences between the two groups in the postoperative total HOAs (mean difference −0.04; 95% CI, −0.23 to 0.14; p = 0.63), spherical (mean difference 0.00; 95% CI, −0.08 to 0.09; p = 0.93), and coma (mean difference −0.06; 95% CI, −0.14 to 0.03; p = 0.20) aberrations.

**Conclusions:**

According to the meta-analysis, wavefront-guided PRK offered no advantage in efficacy, predictability, or safety measures over non-wavefront-guided PRK, although it may have induced fewer trefoil aberrations.

## Introduction

Today, excimer refractive surgery is the treatment of choice for the correction of low to moderate myopia. [1]–[4] However, irregularities in the optical system after refractive surgery can cause subjective complaints, such as halos and glare, that are attributed to an increase in higher-order aberrations (HOAs). [5], [6] This explains why nearly 30% of patients report night-vision problems after successful laser refractive surgery. [7], [8].

Wavefront-guided ablation was introduced to better control aberrations caused by refractive surgery. [9], [10] However, the effectiveness of wavefront-guided ablation has been questioned. Studies comparing wavefront-guided ablation and conventional treatment report inconsistent results, ranging from a significant reduction in preexisting aberrations to no difference to deterioration in preexisting HOAs in wavefront-guided-treated eyes. [11]–[14] According to the meta-analysis, wavefront-guided laser in situ keratomileusis (LASIK) did not offer any advantage in efficacy, predictability, or safety measures over non-wavefront-guided LASIK, although it induced fewer total HOAs. [15] However, as far as we can ascertain, there have so far been no clinical studies that have compared these visual, refractive, aberrometric outcomes between wavefront-guided and non-wavefront-guided photorefractive keratectomy (PRK) using meta-analysis. To determine whether wavefront-guided ablation shows any advantages over non-wavefront-guided alternatives in PRK, we performed a meta-analysis of all published randomized controlled trials (RCTs) of wavefront-guided laser treatments. We also compared the efficacy, predictability, safety, and change in optical aberrations between wavefront-guided and non-wavefront-guided PRK treatments.

## Materials and Methods

In the following databases, data source articles limited to RCTs and published between January 2000 and the end of December 2013 were examined: PubMed (Medline), EMBASE, and Cochrane Central Register of Controlled Trials. Photorefractive keratectomy is the term for sensitive searches. Also, the reference lists of every primary article and all previous systematic reviews were scrutinized for information about additional trials. The searches were not restricted to a specific language.

### Trial Selection

First, 2 reviewers (H.K., K.H.) independently assessed studies for possible eligibility at the title and/or abstract level. The inclusion criteria were met in patients randomly assigned prospectively to refractive error correction with a wavefront-guided laser treatment or a non-wavefront-guided laser treatment. A minimum follow-up of 3 months was required.

### Data Extraction

Two independent reviewers (H.K., K.K.) extracted data from the included trials using a standardized form. The data of interest for each clinical outcome were extracted as follows:

1. Efficacy: the number of eyes postoperatively achieving an uncorrected distance visual acuity (UDVA) of 20/20 or better.
2. Predictability: the number of eyes achieving a postoperative spherical equivalent (SE) within ±0.50 diopter (D) of the intended target.
3. Safety: the number of eyes that lost 2 or more lines of postoperative corrected distance visual acuity (CDVA) relative to the preoperative CDVA [16]–[18].
4. Higher-order aberrations: the root mean square (RMS) values of the total HOAs, spherical, coma, and trefoil aberration.

The data extracted from each study were the numbers of eyes in the wavefront-guided and non-wavefront-guided groups according to the criteria stated above for the final follow-up visit. The sample size and the follow-up period were also recorded. The corresponding authors of the individual trials were also contacted for unpublished information.

The quality of each trial was assessed using the Jadad et al. [19] score with a scale of 0 to 5. Each trial was assessed by 3 main aspects of study design: randomization, masking, and participant withdrawals/dropouts. Trials with a score higher than 3 were considered to be of high quality.

### Statistical Analysis

For efficacy, predictability, and safety, the data in each study were tabulated into 2 × 2 tables and the odds ratio (OR) and 95% confidence intervals (CIs) of the results between wavefront-guided treatments and non-wavefront-guided treatments were compared. The pooled OR and 95% CI were also determined according to a previously described methodology. [20] For HOAs, the statistical option used for meta-analysis was the weighted mean difference for comparing the mean postoperative RMS ± standard deviation (SD) values between the 2 groups. Eyes were divided into 2 subgroups on the basis of the preoperative total HOA values. Heterogeneity was also assessed, and an I^2^ value greater than 50% was considered significant. In this instance, a random-effects model was used because it gives a more conservative estimate and is less influenced by the weighting of each study. [21] When the level of heterogeneity was less than 50%, a fixed-effect model was used. Publication bias was assessed visually with a funnel plot. [22] Meta-analysis was performed with Revman software (version 5.2, Information Management Systems Group, Cochrane Collaboration).

## Results

### Results of Search

**Figure 1:**
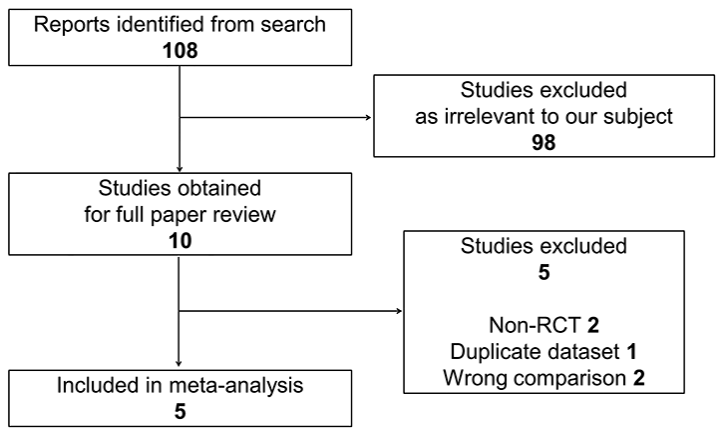
Flow of trial selection. RCTs = randomized controlled trials.

There were 108 articles relevant to the search terms. Ninety-eight studies were excluded after abstract evaluation. Of 10 publications, [23]–[32] which were initially considered potentially relevant, five were excluded because they did not meet the predefined inclusion criteria (Figure 1). [24], [26], [27], [29], [31] The other five prospective RCTs were included in this meta-analysis. [23], [25], [28], [30], [32].

### Characteristics and Quality of Trials

Table 1 shows the main characteristics and quality score of the included trials. Three [28], [30], [32] of the 5 trials were unilateral-eye studies; eyes were matched for SE, and age matching and sex matching was not required. The other 2 studies [23], [25] used 2 different populations in 2 of the trials that matched groups for age and SE. Four studies compared wavefront-guided PRK and conventional PRK [23], [25], [28], [32], and the other study compared wavefront-guided PRK and wavefront-optimized PRK [30]. No study compared wavefront-guided PRK and topography-guided PRK. All the 5 trials were deemed to be of high quality (Jadad score ≥3).

**Table 1:** Characteristics and quality of included trials evaluating wavefront-guided versus non-wavefront-guided photorefractive keratectomy.

Study* (Year)	Country	Score**	Mean Preop SE (D)		Platform	
			WG-PRK	NWG-PRK	WG-PRK	NWG-PRK
Mifflin [32] (2012)	USA	4	−3.22±1.86	−3.2±1.72	Visix Cutsom Vue	Visix S4, conv.
Moshirfar [30] (2011)	USA	3	−3.34±1.75	−3.26±1.82	Visix Cutsom Vue	Wavelight Allegretto
Karimian [28] (2010)	Iran	5	−4.92±1.60	−4.92±1.60	Technolas 217z Zyoptic	Technolas 217z, conv.
Mastropasqua [25] (2006)	Italy	3	−2.25±0.76	−2.35±1.10	Technolas 217z Zyoptic	Technolas 217z, conv.
Mastropasqua [23] (2004)	Italy	3	−4.39±1.31	−4.08±0.97	Meditec MEL 70, WASCA	Meditec MEL 70, conv.

SE = spherical equivalent; WG = wavefront-guided; NWG = non-wavefront-guided; PRK = photorefractive keratectomy.

*First author; **Jadad scores.

### Efficacy

**Figure 2:**
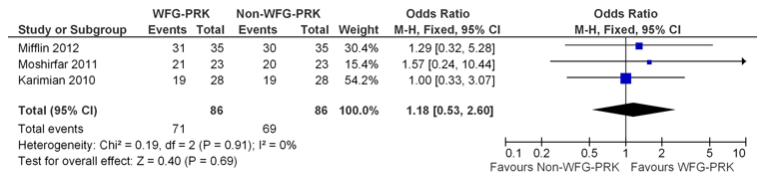
Forest plot comparing efficacy of WFG-PRK and Non-WFG-PRK. Each study is represented by a square at the point of treatment effect, and the area of the square is proportional to the weighting of that study in the analysis. A horizontal line on either side of each square represents the CI. The diamond at the bottom of the plot represents the overall treatment effect determined by the position at the center of the diamond and the CI of the combined data conveyed by the width of the diamond. WFG-PRK =  wavefront guided photorefractive keratectomy, Non-WFG-PRK =  non-wavefront guided photorefractive keratectomy, M-H = Mantel-Haenszel, CI = confidence interval, Chi^2^ = chi-square statistic, df = degrees of freedom, I^2^ = I-square heterogeneity statistic, Z = Z-statistic.

Meta-analysis in relation to efficacy was performed for 3 of the 5 studies because 2 studies [23], [25] reported no proportion of eyes with a UDVA of 20/20 or better. The efficacy Forest plot showed an equivalent treatment efficacy between wavefront-guided PRK and non-wavefront-guided PRK (OR, 1.18; 95% CI, 0.53–2.60; p = 0.69) (Figure 2).

### Predictability

**Figure 3:**
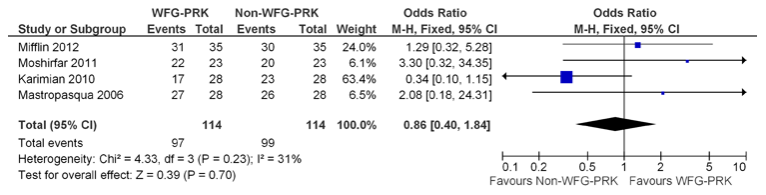
Forest plot comparing predictability of WFG-PRK and Non-WFG-PRK. WFG-PRK =  wavefront guided photorefractive keratectomy, Non-WFG-PRK =  non-wavefront guided photorefractive keratectomy, M-H = Mantel-Haenszel, CI = confidence interval, Chi^2^ = chi-square statistic, df = degrees of freedom, I^2^ = I-square heterogeneity statistic, Z = Z-statistic.

Meta-analysis in relation to predictability was performed for 4 of the 5 studies because one study [23] did not include data on predictability. The predictability Forest plot showed equivalent predictability between wavefront-guided PRK and non-wavefront-guided PRK (OR, 0.86; 95% CI, 0.40–1.84; p = 0.70) (Figure 3).

### Safety

Data for this outcome were collected from 2 trials. [30], [32] No patient lost 2 or more lines of CDVA; hence meta-analysis was not applicable. Table 2 shows the clinical data in the 5 studies with regard to safety.

**Table 2:** Eyes losing or gaining lines in Snellen CDVA.

	Number (%)							
	Lost≥2 Lines		Lost 1 Line		No Change		Gained≥1 Lines	
Study*	WG-PRK	NWG-PRK	WG-PRK	NWG-PRK	WG-PRK	NWG-PRK	WG-PRK	NWG-PRK
Mifflin [32]	0	0	4 (11.4)	2 (5.7)	18 (51.4)	13 (37.1)	13 (37.1)	20 (57.1)
Moshirfar [30]	0	0	1 (4.3)	1 (4.3)	16 (69.6)	16 (69.6)	6 (26.1)	6 (26.1)
Karimian [28]	NA	NA	NA	NA	NA	NA	NA	NA
Mastropasqua [25]	NA	NA	NA	NA	NA	NA	NA	NA
Mastropasqua [23]	NA	NA	NA	NA	NA	NA	NA	NA

CDVA = corrected distance visual acuity; NA = data not available; WG = wavefront-guided; NWG = non-wavefront-guided; PRK = photorefractive keratectomy.

*First author.

### Higher-Order Aberrations

**Figure 4:**
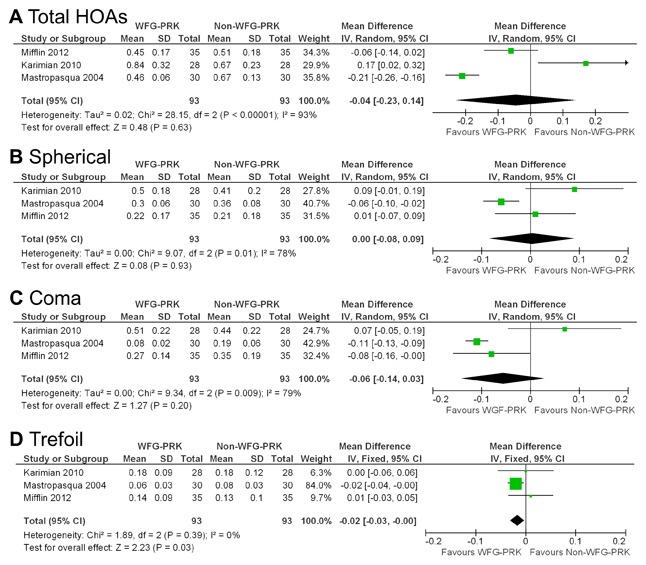
Forest plot comparing HOAs outcomes after treatment with WFG-PRK and Non-WFG-PRK. **A**, Total HOAs. **B**, Spherical aberration. **C**, Coma aberration. **D**, Trefoil aberration. HOAs = higher-order aberrations, WFG-PRK =  wavefront guided photorefractive keratectomy, Non-WFG-PRK =  non-wavefront guided photorefractive keratectomy, IV = inverse variance, CI = confidence interval, Tau^2^ = tau-square statistic, Chi^2^ = chi-square statistic, df = degrees of freedom, I^2^ = I-square heterogeneity statistic, Z = Z-statistic.

Meta-analysis in relation to total HOAs, and to spherical, coma, and trefoil aberrations was conducted in 3 of the 5 studies. Of the 2 omitted studies, one [25] reported results on preoperative total HOAs, but the postoperative values for each group were not reported. One study [30] reported the HOA data in the figures, but the aberratios values for each group were not shown in the text. The total HOAs, spherical, and coma aberration Forest plots showed no significant differences in these values after treatment between wavefront-guided PRK and non-wavefront-guided PRK (Figure 4 A, B, and C). The trefoil aberration Forest plots showed that the increase in trefoil aberration in patients who had wavefront-guided PRK was less than that in those who had non-wavefront-guided PRK (weighted mean difference = −0.02; 95% CI, −0.03 to −0.00; p = 0.03) (Figure 4 D). Table 3 shows the clinical data in the 5 HOA studies.

**Table 3:** Preoperative and postoperative total HOAs values by group.

		Preop total HOAs		Postop total HOAs	
Study*	Pupil size (mm)	WG-PRK	NWG-PRK	WG-PRK	NWG-PRK
Mifflin [32]	6.0	0.38±0.13	0.33±0.13	0.45±0.17	0.51±0.18
Moshirfar [30]	6.0	NA	NA	NA	NA
Karimian [28]	6.0	0.33±0.09	0.34±0.08	0.84±0.32	0.67±0.23
Mastropasqua [25]	6.0	0.35±0.10	0.32±0.14	NA	NA
Mastropasqua [23]	5.0	0.27±0.10	0.28±0.09	0.46±0.06	0.67±0.13

HOAs = higher-order aberrations; WG = wavefront-guided; NWG = non-wavefront-guided; PRK = photorefractive keratectomy; NA = data not available.

*First author.

**Figure 5:**
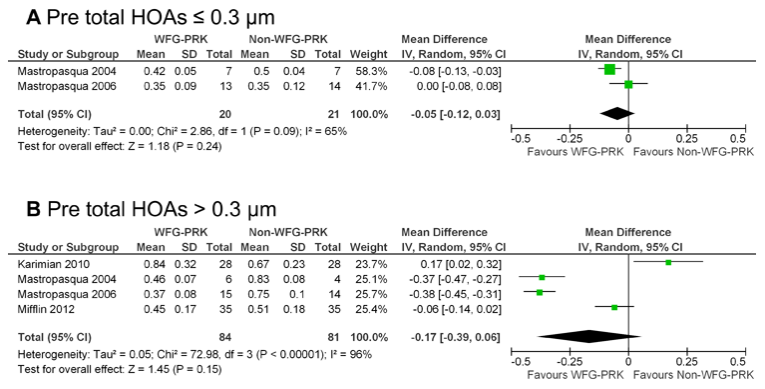
Forest plot comparing total HOAs outcomes after treatment with WFG-PRK and Non-WFG-PRK in patients divided into 2 groups based on preoperative total HOAs. **A**, Preoperative total HOAs ≤0.3 µm. **B**, Preoperative total HOAs >0.3 µm. HOAs = higher-order aberrations, WFG-PRK =  wavefront guided photorefractive keratectomy, Non-WFG-PRK =  non-wavefront guided photorefractive keratectomy, IV = inverse variance, CI = confidence interval, Tau^2^ = tau-square statistic, Chi^2^ = chi-square statistic, df = degrees of freedom, I^2^ = I-square heterogeneity statistic, Z = Z-statistic.

Two studies [23], [25] reported data with preoperative total HOA errors ≤0.3 µm. Analysis of these data revealed that the difference between the two groups was not statistically significant (weighted mean difference = −0.05; 95% CI, −0.12 to 0.03; p = 0.24) (Figure 5 A). Four studies [23], [25], [28], [32] reported data with preoperative total HOA errors of more than 0.3 µm. Analysis of these data revealed that the difference between the two groups was not statistically significant (weighted mean difference = −0.17; 95% CI, −0.39 to 0.06; p = 0.15) (Figure 5 B).

### Complications

None of patients in either group showed evidence of corneal haze in both groups at the last follow-up visit [23], [25], [28], [32].

### Publication Bias

**Figure 6:**
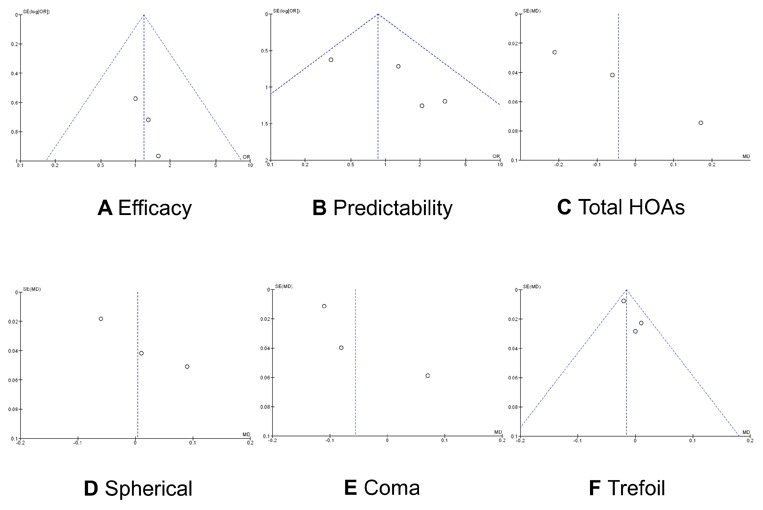
Funnel plots showing the distribution between studies comparing each outcome. **A**, Efficacy, **B**, Predictability, **C**, Total HOAs. **D**, Spherical aberration. **E**, Coma aberration. **F**, Trefoil aberration.

The publication bias was independently assessed graphically for each clinical outcome using funnel plots (Figure 6). For efficacy and predictability, each study was plotted with the OR against the standard error as a measure of weighting. For aberrations, each study was plotted with the weighted mean difference against the standard error as a measure of weighting. There was an almost equal distribution between studies with a low and high OR/weighted mean difference and a low and high standard error for efficacy, predictability, and trefoil aberration.

## Discussion

This meta-analysis provides evidence that wavefront-guided and non-wavefront-guided PRK approaches can provide similar results in terms of visual outcomes (efficacy, predictability, and safety). Our results agree with those of Fares et al. [15], who also noted these outcomes after wavefront-guided LASIK using meta-analysis. To our knowledge, this is the first published study to compare the visual outcomes between wavefront-guided and non-wavefront-guided PRK techniques using meta-analysis. Netto et al. [33] reviewed the benefits and limitations of wavefront-guided PRK. They concluded that, while wavefront-guided refractive surgery provides excellent results, evidence is limited that it outperforms the conventional technique, which incorporates broad ablation zones, smoothing to the periphery, eye-trackers, and other technological refinements.

Although PRK is currently not a gold standard of corneal refractive surgery, it offers some advantages over LASIK, in terms of less induction of higher-order aberrations [34], less induction of refractive regression [35], better biomechanical stability [36], and no risk of flap-related complications [37]. Therefore, PRK is still one of the viable surgical options for the correction of refractive errors.

The key indicator of efficacy of any given refractive surgical procedure is visual acuity. It is important that visual acuity should be specifically recorded for each time point, and that results should refer to the exact line seen by the patient on the Snellen acuity chart. Such detailed information is critical when outcomes in different studies are compaied. [38]–[40] Throughout our review, we noticed that authors had presented their outcome data in diverse ways. However, 3 of 5 studies reported the UDVA to be 20/20 or better. Thus, the efficacy in our study was evaluated only at 20/20 or better. The percentage of eyes with a UDVA of 20/20 or better at the last follow-up visit was 82.6% in the wavefront-guided group and 80.2% in the non-wavefront-guided group. However, this difference was not statistically significant.

Predictability data was reported by 4 of the 5 studies that qualified for inclusion in our study. The percentage of eyes with an SE of ±0.50 D at the last follow-up visit was 85.1% in the wavefront-guided group and 86.8% in the non-wavefront-guided group. However, this difference was not statistically significant.

Safety is also an important parameter when refractive surgery outcomes are reported, and it should be expressed in terms of the change in the Snellen lines of CDVA. Although the safety of both wavefront-guided PRK and non-wavefront-guided PRK has been reported, only 2 of the 5 RCTs included in our meta-analysis reported their safety criteria. It is important that more attention be paid to reporting safety criteria in studies of refractive surgery outcomes.

HOA data was reported by 3 of the 5 studies that qualified for inclusion in our study. We found no significant difference in the postoperative total HOAs, spherical, or coma aberration between wavefront-guided and non-wavefront-guided groups. However, there was a significant difference in postoperative trefoil aberration between the two groups. As far as we can ascertain, this is also the first study to assess the HOAs including spherical, coma, and trefoil aberrations after wavefront-guided and non-wavefront-guided PRK techniques using meta-analysis. Overall wavefront-guided PRK had fewer induced HOAs than non-wavefront-guided PRK, although our meta-analysis showed no significant difference. For eyes with preoperative total HOA values of ≤0.3 µm, the mean postoperative total HOA did not differ significantly between the two groups. On the one hand, in cases with preoperative total HOA values of >0.3 µm, wavefront-guided PRK resulted in a slightly better postoperative aberration profile, suggesting that in eyes with high preexisting HOAs, wavefront-guided treatment may have advantages over non-wavefront-guided treatment. With regard to wavefront-guided LASIK, meta-analysis showed that wavefront-guided technology induced a smaller increase of postoperative wavefront-error compared to non-wavefront-guided technology, particularly in patients with higher preoperative HOAs. [15] The induction of HOAs may lead to a deterioration in visual performance and subsequent patient dissatisfaction. As conventional PRK technique requires more laser ablation in high myopic eyes, the cornea becomes more oblate, resulting in more surgically induced HOAs, especially spherical aberrations. Wavefront-guided treatment has been reported to be more effective in reducing the induction of HOAs than conventional treatment.

This meta-analysis has several limitations that should be taken into account when its results are considered. First, the follow-up duration reported in these trials was limited. All the five studies reported data for less than 1 year, limiting the value of conclusions concerning the stability and long-term regression. Additionally, the recovery of corneal innervation and the restoration of a normal tear film and the ocular surface may take longer than 12 months. [41], [42] There is evidence that increases in higher order aberrations resulted from increased tear film and ocular surface irregularity. [43] Second, the small number of cases per trial and in total give these analyses low power. Subgroup analysis related to the degree of myopia was not possible because there was insufficient data. Nevertheless, this meta-analysis provides more powerful evidence than the individual reports alone, and we are unaware of any other similar meta-analyses. Third, we could include only data from published articles, and it is possible that bias is introduced if studies with small or reverse effects exist but have not been published. However, our asymmetrical funnel plots for efficacy, predictability, and trefoil aberration indicate that publication bias may be ruled out.

In summary, it can be assumed from this meta-analysis that wavefront-guided and non-wavefront-guided procedures have comparable efficacy, predictability, and safety for PRK. In addition, wavefront-guided PRK may induce fewer trefoil aberrations than non-wavefront-guided PRK, which might make it a better choice for wavefront custom ablation, although both approaches result in similar wavefront outcomes for total HOAs, spherical, and coma aberrations. Long-term follow-up trials with large patient populations are needed to determine the relative merits wavefront-guided and non-wavefront-guided PRK using current-day equipment and techniques.

## Supporting Information

Checklist S1 **PRISMA checklist.**
(DOC)
